# *Scutellaria baicalensis* Extract Protects Against Cerebral Ischemia-Reperfusion Injury in Male Rats by Inhibiting Ferroptosis via the PI3K/AKT Pathway

**DOI:** 10.3390/nu18132073

**Published:** 2026-06-24

**Authors:** Mengxuan Zhang, Xueao Chen, Chenhuan Shentu, Dongdong Jin, Jiaying Zhu, Chendao Ruan, Mingjiang Mao, Xiaofeng Yuan

**Affiliations:** 1 The School of Life Sciences, Zhejiang Chinese Medical University, Hangzhou 310053, China; 19012765755@163.com (M.Z.); 15394341319@163.com (X.C.); 15267009322@163.com (C.S.); jindd_huier@sina.com (D.J.); 18868429280@163.com (J.Z.); 2 Department of Environmental Engineering, College of Environmental and Resource Science, Zhejiang University, Hangzhou 310058, China; 12514001@zju.edu.cn

**Keywords:** *Scutellaria baicalensis* extract, cerebral ischemia reperfusion injury, ferroptosis, PI3K/AKT pathway

## Abstract

**Background:** *Scutellaria baicalensis* (Scu) extract has been traditionally used in the treatment of stroke-related syndromes, yet its underlying molecular mechanisms, particularly those involving ferroptosis, remain to be fully elucidated. **Purpose:** This study aims to validate the hypothesis that Scu extract improves cerebral ischemia-reperfusion injury (CIRI) by inhibiting ferroptosis through the PI3K/AKT signaling pathway. **Methods:** This study employed middle cerebral artery occlusion (MCAO) in male Sprague-Dawley (SD) rats and oxygen–glucose deprivation/reoxygenation (OGD/R) models to evaluate the protective effects of Scu extract against CIRI. Multiple approaches were integrated to elucidate the underlying mechanisms. Furthermore, a range of experimental techniques, including neurological function assessment, TTC staining, histopathological analysis, biochemical assays, qPCR, transmission electron microscopy (TEM), reactive oxygen species (ROS) detection, Western blotting, and immunofluorescence, were used to comprehensively validate its neuroprotective effects. **Results:** Scu extract significantly improved neurological outcomes and attenuated brain injury in MCAO rats. Proteomic analysis revealed significant enrichment of ferroptosis-related pathways, which was supported by reduced mitochondrial damage, decreased iron accumulation, and restoration of the SLC7A11/GPX4 axis. Subsequently, UPLC/Q-TOF-MS analysis revealed that four major bioactive components were absorbed in MCAO rats. KEGG pathway analysis based on network pharmacology further indicated that the PI3K/AKT signaling pathway is a key regulatory target. Notably, pharmacological inhibition of PI3K with LY294002 markedly abolished the anti-ferroptotic effects of Scu extract, which was further confirmed in vitro. **Conclusions:** This study demonstrates that Scu extract confers neuroprotection against CIRI in MCAO rats potentially through inhibiting ferroptosis via activation of the PI3K/AKT pathway.

## 1. Introduction

Ischemic stroke (IS) is a major cerebrovascular disease with high mortality and disability. Its underlying mechanism involves cerebral thrombosis, which leads to interrupted cerebral blood flow, thereby triggering localized ischemic necrosis of brain tissue and irreversible neuronal damage [1,2,3]. Currently, treatment of IS aims to restore cerebral blood flow as early as possible to salvage the ischemic penumbra. However, reperfusion is frequently accompanied by complications, among which CIRI is recognized as the most severe and unavoidable one [4]. From a mechanistic perspective, the sudden surge of oxidative stress triggered upon reperfusion is considered the key initiating factor for secondary neuronal damage. When cerebral blood supply is restored, surviving neurons are subjected to a secondary oxidative stress insult; excessive accumulation of lipid peroxidation has been shown to induce iron-dependent neuronal death, thereby further exacerbating CIRI [5].

Among the various forms of regulated cell death, ferroptosis has emerged as a key driver of iron-dependent neuronal death in CIRI. Unlike apoptosis and necroptosis, ferroptosis is a distinct, iron-dependent form of regulated cell death primarily driven by excessive lipid peroxidation. Apoptosis is caspase-dependent and characterized by cell shrinkage, chromatin condensation, and apoptotic body formation without triggering inflammation. Necroptosis is mediated by the RIPK1/RIPK3-MLKL signaling axis, leading to cell swelling, membrane rupture, and release of damage-associated molecular patterns (DAMPs) [6,7]. In contrast, ferroptosis does not involve caspase activation or RIPK signaling; its hallmark features include mitochondrial shrinkage, cristae reduction or loss, with intact nuclei. Ferroptosis is closely linked to disturbances in redox balance and iron homeostasis [8]. Mitochondria are key organelles involved in ferroptosis. In CIRI, Fe^2+^ overload induces mitochondrial dysfunction and produces large amounts of ROS, thereby exacerbating lipid peroxidation [9]. A central regulator of this process is glutathione peroxidase 4 (GPX4), which detoxifies lipid peroxides to preserve membrane stability. The activity of GPX4 relies on the Xc^−^–glutathione (GSH)–GPX4 system, where cystine uptake via system Xc^−^ supports intracellular GSH synthesis, enabling GPX4 to function effectively. During this period, impairment of this antioxidant system—manifested by GSH depletion, reduced GPX4 activity, and elevated malondialdehyde (MDA) levels—leads to excessive lipid peroxidation and ultimately triggers ferroptotic cell death. However, under CIRI conditions, enhanced iron influx combined with impaired storage and export results in Fe^2+^ accumulation, which promotes ROS generation via the Fenton reaction. This cascade amplifies oxidative damage and ferroptosis. Mitochondrial dysfunction and Fe^2+^ accumulation mutually reinforce each other, forming a vicious cycle [3,10,11,12]. Therefore, modulation of both the Xc^−^–GSH–GPX4 axis and iron metabolism represents a potential therapeutic approach for CIRI.

Recent studies have shown that targeting upstream signaling pathways associated with ferroptosis to suppress oxidative stress amplification and abnormal lipid peroxidation represents a crucial strategy for interrupting the pathological cascade of CIRI [13,14,15]. Due to its critical role in cell survival, metabolic homeostasis, and programmed cell death regulation, the phosphoinositide 3-kinase/protein kinase B (PI3K/AKT) signaling pathway has gradually been recognized as a key molecular mechanism regulating ferroptosis in CIRI [16]. Previous studies have demonstrated that the PI3K/AKT pathway exerts neuroprotective effects by regulating key nodes in ferroptosis and exhibits potential therapeutic value in preventing and treating CIRI [17]. Within the PI3K family, Class I PI3K is most closely associated with the regulation of ferroptosis. Its activation is typically triggered by extracellular signals, such as growth factors released following cerebral ischemia, which stimulate receptor tyrosine kinases and subsequently promote AKT phosphorylation. Activated AKT suppresses ferroptosis induced by iron overload and lipid peroxidation by regulating autophagy homeostasis, inhibiting inflammatory responses, and maintaining the expression and activity of GPX4—a key ferroptosis-related protein—thereby mitigating brain tissue damage caused by CIRI [18,19,20]. Thus, investigating the involvement of the PI3K/AKT pathway in ferroptosis regulation may provide novel insights into strategies for CIRI management.

In the theoretical framework of traditional Chinese medicine, IS is classified under the category of “Zhongfeng” (stroke). Its pathogenesis is primarily attributed to liver and kidney yin deficiency, insufficiency of qi and blood, and dysfunction of the zang-fu organs, which consequently lead to the endogenous generation of phlegm turbidity and blood stasis. In addition, invasion of exogenous wind pathogens may promote the interaction between phlegm and stasis, resulting in obstruction of cerebral collaterals and ultimately triggering stroke [21,22]. Through long-term clinical practice, TCM has accumulated extensive experience in the prevention and treatment of stroke, leading to the development of numerous effective prescriptions, among which Scu is one of the most frequently used herbs. Scu, derived from the dried root of *Scutellaria baicalensis* Georgi (Lamiaceae), has long been used in traditional medicine for heat-clearing, detoxification, and regulating internal balance, including blood cooling and miscarriage prevention. Classical materia medica texts document its therapeutic applications in conditions such as heat-induced jaundice, dysentery, edema, hematochezia, and ulcerative lesions [23,24]. It has been officially listed in the Chinese Pharmacopoeia (2025 edition). Clinically, Scu is frequently prescribed for stroke syndromes characterized by hyperactivity of liver yang and accumulation of phlegm-heat [25]. In classical prescriptions, *Xiaoxuming decoction*, documented in *Essential Prescriptions for Emergencies* by Sun Simiao during the Tang Dynasty, includes Scu as a key component and is widely used to treat stroke-related symptoms, such as facial paralysis and hemiplegia, further underscoring its important role in stroke management [26]. Importantly, the traditional use of Scu in stroke-related syndromes, particularly those characterized by phlegm-heat and internal imbalance, suggests its potential involvement in pathological processes such as oxidative stress and lipid peroxidation, which are closely associated with ferroptosis [27]. Moreover, previous pharmacological studies have demonstrated that Scu exhibits anti-inflammatory, antioxidant, and neuroprotective effects in cerebrovascular diseases, thereby providing scientific support for its traditional application in stroke treatment [28,29]. In recent years, increasing evidence has demonstrated the neuroprotective effects of Scu and its active constituents in ischemic brain injury. Fang et al. [30] reported that Scu polyphenols inhibit ferroptosis by regulating the SIRT6/FOXA2/SLC7A11 signaling axis, thereby maintaining neuronal redox homeostasis and attenuating cerebral ischemic injury. Similarly, Li et al. [5] demonstrated that baicalein, a major active component of Scu, suppresses ferroptosis and alleviates CIRI by modulating GPX4/ACSL4/ACSL3-related pathways. Taken together, these findings suggest that Scu may exert neuroprotective effects against CIRI through the regulation of ferroptosis-related signaling pathways, thereby providing a mechanistic basis for its traditional application in stroke treatment.

In summary, although Scu has shown neuroprotective potential in ischemic brain injury, whether it protects against CIRI by regulating ferroptosis remains insufficiently understood. In particular, the upstream signaling mechanisms linking Scu extract to ferroptosis inhibition have not been fully clarified. Therefore, this study aimed to investigate the protective effects of Scu extract against CIRI and to determine whether the PI3K/AKT pathway mediates its regulation of ferroptosis. To this end, rat MCAO and OGD/R-induced PC12 cell models were used, combined with proteomics, network pharmacology, molecular docking, and experimental validation. These findings are expected to deepen the understanding of ferroptosis-related mechanisms in CIRI and provide a scientific basis for the development of Scu extract as a potential therapeutic strategy for IS.

## 2. Materials and Methods

### 2.1. Preparation of Scu Extract

Dried *Scutellaria baicalensis* root (Batch No. 20240825, Zhejiang Tiandao Pharmaceutical Co., Ltd., Hangzhou, China) was ground into powder and passed through a sieve. An accurately weighed portion (50 g) of the powder was added to a round-bottom flask, and later, 75% (*v*/*v*) ethanol (750 mL) was added. Thereafter, this resultant mixture was macerated for 30 min under ambient temperature to ensure complete wetting and facilitate the extraction of active components. Subsequently, extraction was carried out for 1 h of reflux. Afterwards, the hot extract was immediately filtered, followed by re-extraction of the residue under identical conditions. Filtrates were then pooled for concentration at reduced pressure with the rotary evaporator (RE-52AA, Yarong, Tai’an, China) to remove the solvent, yielding an extract equivalent to 0.5 g/mL of crude material. The concentrated extract was thoroughly mixed and divided into identical parts in sterile centrifuge tubes before storage at −80 °C until further use.

### 2.2. UPLC/Q-TOF-MS Analysis

#### 2.2.1. Preparation of Blank Serum and Scu-Containing Serum

After one week of acclimatization, rats were randomly assigned to the saline or Scu group (3 g·kg^−1^, *n* = 3 per group) and treated by intragastric administration once daily for 7 days. Two hours after the final dose, rats were anesthetized, and blood was collected from the abdominal aorta. Serum was obtained by centrifugation, filtered through a 0.22 μm membrane, aliquoted, and stored at −80 °C until use.

#### 2.2.2. Scu Extract and Plasma Sample Preparation for UPLC/Q-TOF-MS Analysis

For Scu extract analysis, 0.6 mL of the extract prepared in Section 2.1 was mixed with 0.4 mL of methanol. The resulting mixture was subjected to 15 min of centrifugation at 12,000 rpm to collect the supernatant. After filtration through a 0.45 μm membrane, the filtrate was transferred into a sample vial for subsequent analysis. In addition, 100 μL of plasma from both the normal saline and Scu groups, as described in Section 2.2.1, was transferred into 1.5 mL centrifuge tubes, followed by the addition of 300 μL of methanol. The mixed samples were vortexed for 5 min to precipitate proteins and then centrifuged for 15 min at 12,000 rpm. The collected supernatant was evaporated under a nitrogen atmosphere. After reconstitution in 300 μL of methanol, the residue was vortexed for 2 min and centrifuged again for 10 min at 12,000 rpm to collect the final supernatant for UPLC/Q-TOF-MS analysis (SYNAPT G2-Si, Waters, Milford, MA, USA). Chromatographic conditions were set as previously described by Cao et al. [31].

### 2.3. Animals and Corresponding Treatments

Male Sprague-Dawley (SD) rats (weighing 260 ± 20 g) were purchased from BK Experimental Animal Co., Ltd. (Shanghai, China) and housed at Zhejiang Chinese Medical University. Rats were housed under controlled conditions (23 ± 3 °C, 50–60% humidity, 12 h light/dark cycle). All procedures were approved by the Scientific Ethics and Safety Committee of Zhejiang Chinese Medical University (Approval No.: IACUC-20241028-16, Hangzhou, China). Animal experiments were conducted in two parts. After one week of acclimatization, rats were randomly assigned to five groups: control, MCAO, L-Scu (1.5 g/kg), H-Scu (3 g/kg), and Nimodipine (20 mg/kg). In the second part, rats were divided into five groups: control, MCAO, H-Scu (3 g/kg), LY294002 (10 mg/kg), and LY294002 + H-Scu (10 mg/kg LY294002 + 3 g/kg Scu extract).

### 2.4. Establishment of MCAO Model, Neurological Assessment, and TTC Staining

MCAO procedures, neurological assessments, and TTC staining were performed as previously described [32]. Briefly, after 12 h of fasting (with free access to water), rats were anesthetized with an intraperitoneal injection of Telazol and placed in the supine position. The left common carotid artery (CCA), external carotid artery (ECA), and internal carotid artery (ICA) were exposed. The distal end of the ECA was ligated. After clamping the CCA, a small incision was made in the ECA, and a nylon monofilament suture was inserted through the ECA and advanced into the ICA to occlude the origin of the middle cerebral artery. Following 60 min of ischemia, the suture was withdrawn to allow reperfusion. In the control group, the arteries were isolated without suture insertion. The mortality rate of MCAO-operated rats was approximately 20–30%.

Neurological deficits were evaluated on days 1, 4, and 7 post-surgery using the Zea Longa 5-point scoring system (0, no deficit; 1, failure to fully extend the left forepaw; 2, circling to the left; 3, falling to the left; 4, loss of spontaneous walking with decreased consciousness) [33]. Only animals with scores ranging from 1 to 3 were considered successfully modeled and included in subsequent experiments. Neurological assessments were performed in a double-blind manner by two independent investigators.

At the end of the treatment period, rats were euthanized, and their brains were rapidly removed and frozen at −80 °C for 10 min. Coronal sections of 2 mm thickness were prepared. Brain slices were incubated in 2% TTC solution at 37 °C in the dark for 15 min. Viable tissues were stained red, while infarcted areas remained white. The infarct volume percentage was calculated using ImageJ software v.1.53a.

### 2.5. Pathological Staining

Brain tissues were processed for H&E or Nissl staining as previously described [31]. Brain tissues were fixed in 4% paraformaldehyde at 4 °C for 24 h, dehydrated through a graded ethanol series, cleared in xylene, and embedded in paraffin, then sectioned into 4 μm thick slices. For H&E staining, sections were deparaffinized, rehydrated, stained with hematoxylin for 5 min, differentiated in 1% hydrochloric acid alcohol for 15 s, counterstained with eosin for 1–2 min, dehydrated, cleared, and mounted with neutral balsam. For Nissl staining, sections were deparaffinized, rehydrated, stained with 0.1% cresyl violet solution at 37 °C for 10–15 min, differentiated in 95% ethanol for a few seconds, dehydrated, cleared, and mounted with neutral balsam. Histopathological changes, neuronal morphology, and Nissl body distribution in the cortex and hippocampal CA1 region were observed under a light microscope. All analyses were performed in a blinded manner.

### 2.6. Proteomics Analysis

The cortical samples were first retrieved from the −80 °C freezer and ground into powder using liquid nitrogen. An appropriate amount of the powdered sample was transferred into 1.5 mL centrifuge tubes, and lysis buffer was added. Ultrasonic lysis was performed on ice for 5 min, and then subjected to 10 min of centrifugation at 15,000 rpm at 4 °C. Next, a BCA assay kit was adopted to determine protein concentration. Finally, protein hydrolysis and desalination were performed based on the determined protein concentration for further analysis. Mass spectrometry raw data were processed using DIA-NN (v1.8.1) employing a library-free method. We generated a spectral library from the Uniprot proteome database (uniprot-proteome_UP000002494_R), which contains 46,069 sequences. From the DIA data, a spectral library was constructed using the Match Between Runs (MBR) option, which was then reanalyzed. Identifications that passed this threshold were used for further quantitative analysis based on the false discovery rate (FDR) at the protein and precursor ion levels. DEPs were identified based on the following criteria: |log_2_FC| ≥ 0.585 and FDR < 0.05. The FDR was calculated using the Benjamini–Hochberg method to correct for multiple comparisons.

### 2.7. TEM

Ultrastructural analysis was performed using TEM as previously reported [34]. Brain tissue blocks of approximately 1 mm^3^ were fixed in 4% glutaraldehyde, post-fixed in 1% osmium tetroxide, dehydrated through a graded acetone series, and embedded in epoxy resin. Ultrathin sections (50–70 nm) were cut, double-stained with uranyl acetate and lead citrate, and then observed under TEM. Mitochondrial ultrastructure in cortical neurons, including swelling, cristae disruption, or loss, was examined.

### 2.8. Biochemical Analysis

An appropriate amount of rat cerebral cortex tissue was collected, homogenized in sample preparation solution under ice-cold conditions, and centrifuged at 4 °C (12,000 rpm) for 10 min. The supernatant was collected to determine MDA levels and SOD and GSH-Px activities (S0131S; S0101S; S0056; Beyotime, Shanghai, China).

### 2.9. Measurement of Total and Ferrous Iron Levels

Cortical tissue was homogenized (10%, *w*/*v*), centrifuged to obtain the supernatant, and total iron (A039-2-1, Nanjing Jiancheng, Nanjing, China) and ferrous iron (E-BC-K773-M, Elabscience, Houston, TX, USA) levels were determined using corresponding assay kits.

### 2.10. Network Pharmacology Analysis

The SMILES structures of the identified compounds were obtained from PubChem, and potential targets were predicted using SwissTargetPrediction, followed by standardization in UniProt. CIRI-related targets were retrieved from GeneCards, and overlapping targets were identified using jvenn. These targets were used to construct a PPI network via STRING (Homo sapiens, confidence ≥ 0.9) and analyzed in Cytoscape 3.10.0. GO and KEGG enrichment analyses were performed using Metascape, and results were visualized as bubble plots.

### 2.11. Molecular Docking

Molecular docking was conducted using Discovery Studio 2019. Ligand structures were obtained from PubChem and converted to MOL2 format, while protein structures of TNF (PDB ID: 7KPA), HSP90AA1 (PDB ID: 8X2R), and EGFR (PDB ID: 8P04) were retrieved from the PDB and prepared in PyMOL v.3.0.4. Docking simulations were performed using the CDOCKER algorithm, and interactions were visualized for analysis. Prior to docking simulations, the reliability of the docking protocol was validated by re-docking the co-crystallized ligand into the binding pocket of each target protein and calculating the root-mean-square deviation (RMSD) between the predicted binding pose and the experimentally determined pose. The RMSD values for all targets were less than 2.0 Å, indicating that the docking protocol was capable of reliably reproducing the native binding conformation.

### 2.12. Cell Viability Assay

The OGD/R model was established as previously described [35]. PC12 cells in the logarithmic growth phase were seeded into 96-well plates at a density of 5 × 10^3^ cells per well. When cell confluence reached 80%, the culture medium was replaced with glucose-free DMEM, and the cells were transferred to a hypoxic chamber gassed with a mixture of 94% N_2_, 5% CO_2_, and 1% O_2_ for 6 h to establish the oxygen-glucose deprivation/reoxygenation (OGD/R) model. Under OGD/R conditions, cells were exposed to different concentrations of blank serum or Scu-containing serum to determine the optimal treatment concentration. Cells were then divided into Control, OGD/R, and Scu groups. Cell viability was measured using a CCK-8 assay (Beyotime, China) at a wavelength of 450 nm.

### 2.13. Measurement of ROS Levels

Intracellular ROS levels were measured as previously described [36]. Intracellular ROS levels were measured using a Reactive Oxygen Species Assay Kit (Beyotime, S0033). PC12 cells were seeded into 96-well plates at a density of 5 × 10^3^ cells/well and divided into five groups: Control, OGD/R, 15%Scu, LY294002, and 15%Scu + LY294002. DCFH-DA was diluted with PBS to a final concentration of 10 μmol/L (1:1000). An appropriate volume of the diluted probe was added to each well, and the cells were incubated for 20 min at 37 °C in the dark. The staining solution was then removed, and the cells were washed three times with PBS to thoroughly eliminate the uninternalized probe. Subsequently, Rosup (diluted 1:1000) was added to stimulate the cells for 20–30 min. DCF green fluorescence was observed and captured using a florescence microscope. All procedures were performed under light-protected conditions.

### 2.14. qPCR Assay

Total RNA from cortical tissue and PC12 cells was reverse-transcribed into cDNA. Quantitative real-time PCR was used to measure TFR1, FTH1, FTL, and FPN1 expression. Expression levels were normalized to β-actin and calculated using the 2^−ΔΔCt^ method. Primer sequences are listed in Table 1.

### 2.15. Western Blotting

Protein from brain cortex tissues were prepared using RIPA lysis buffer (Merck, Darmstadt, Germany, P2714) supplemented with a protease and phosphatase inhibitor cocktail (Beyotime, P1045). Protein concentrations were determined using a BCA protein assay kit (Nanjing Jiancheng, A045-4-2). Equal amounts of denatured protein samples (heated at 100 °C for 5 min) were separated by SDS–PAGE using a one-step PAGE gel preparation kit (EpiZyme, Cambridge, MA, USA, PG212) and subsequently transferred onto PVDF membranes (Tuoran Bio, Wolfsburg, Germany, PVDF45403). The PVDF membranes were activated in methanol for 1 min and equilibrated in pre-chilled transfer buffer for 5 min prior to assembly. After transfer, the membranes were blocked with rapid blocking buffer (Servicebio, Wuhan, China, G2052-500ML) for 10 min at room temperature. The membranes were then incubated overnight at 4 °C with primary antibodies against PI3K (HUABIO, Woburn, MA, USA, ET1608-70, RRID: AB_3069823; diluted 1:1000), AKT (Servicebio, GB15689, RRID: AB_3720808; 1:1000), phosphorylated PI3K (HUABIO, HA721672, RRID: AB_3072785; 1:1000), phosphorylated AKT (HUABIO, ET1607-73, RRID: AB_2940863; 1:1000), GPX4 (Affinity, San Francisco, CA, USA, DF6701, RRID: AB_2838663; 1:1000), and SLC7A11 (Proteintech, Rosemont, IL, USA, 82115-2-RR, RRID: AB_3670516; 1:1000). β-Actin (HUABIO, HA722023; 1:6000) served as the loading control. After three washes with TBST (10 min each), the membranes were incubated with HRP-conjugated secondary antibodies (HUABIO, HA1001; 1:6000) for 2 h at room temperature. Protein bands were visualized using enhanced chemiluminescence (ECL) reagents (Beyotime, P0018S) and images were captured with a chemiluminescence imaging system (Clinx ChemiScope 6200). Band intensities were quantified using ImageJ software (NIH, version 1.53) and normalized to β-actin. All experiments were performed with three independent biological replicates.

### 2.16. Immunofluorescence Staining

Sections were processed for deparaffinization and antigen retrieval, blocked with 5% BSA, and incubated overnight at 4 °C with primary antibodies against SLC7A11 and GPX4. After washing, fluorescent secondary antibodies were used for detection. Following DAPI nuclear counterstaining, photography was performed with the fluorescence microscope. ImageJ was adopted to quantify fluorescence intensities for evaluating SLC7A11 and GPX4 levels. All image quantification was performed by an investigator blinded to the experimental groups.

### 2.17. Statistical Analysis

Data analysis was performed using GraphPad Prism (version 9.5.0, GraphPad Software). Results are expressed as mean ± SD. Group comparisons were conducted using one-way ANOVA, followed by Tukey’s multiple comparison test as the post hoc test. A *p*-value < 0.05 was considered statistically significant.

## 3. Results

### 3.1. Scu Extract Ameliorated Neurological Deficits and Reduced Infarct Volume, Histopathological Injury, and Oxidative Stress

**Figure 1:**
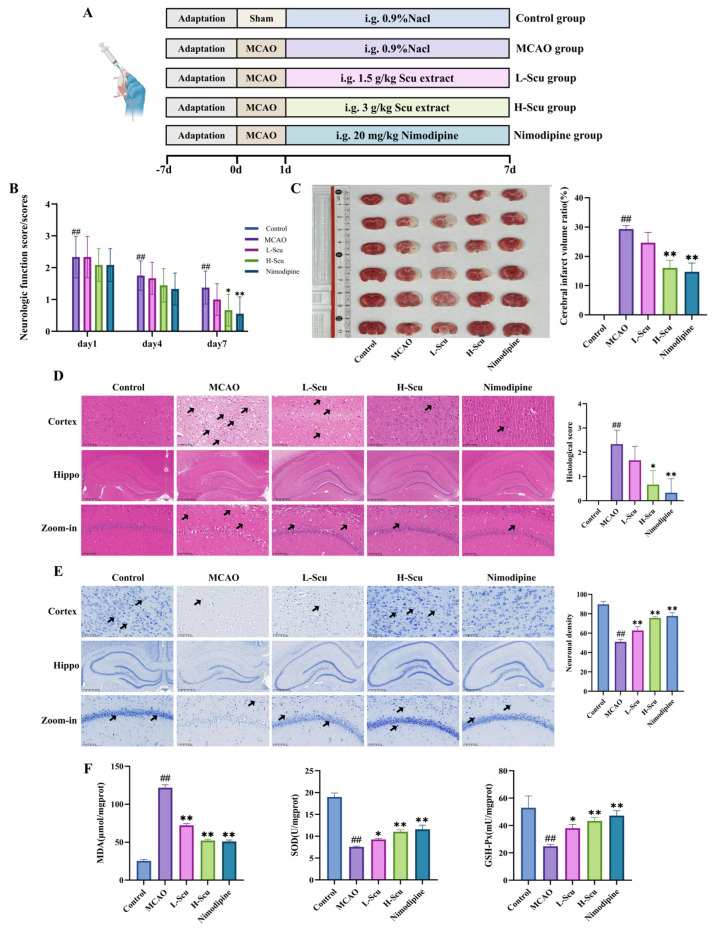
Effects of Scu extract on neurological function, cerebral infarction, histopathological changes, and oxidative stress in MCAO rats. (**A**) schematic illustration of model building and intervention; (**B**) neurological scores; (**C**) TTC staining and cerebral infarction volume ratio; (**D**) representative H&E staining images and histological scores (×200), the black arrow pointed to the damaged neuronal cells; (**E**) representative Nissl staining images and neuronal density (×200), the black arrow pointed to the Nissl body; (**F**) MDA content, SOD and GSH-Px activities. Data are presented as mean ± SD (*n* = 12 for (**B**); *n* = 3 for (**C**–**F**)). ^##^ *p* < 0.01 vs. Control group; * *p* < 0.05, ** *p* < 0.01 vs. MCAO group.

As shown in Figure 1A, rats were randomly assigned to the Control, MCAO, L-Scu, H-Scu, and Nimodipine groups to evaluate the neuroprotective effects of Scu extract. Initially, Scu extract markedly attenuated MCAO-induced neurological deficits and cerebral infarction (Figure 1B,C), highlighting its therapeutic potential. H&E staining (Figure 1D) showed that neurons in the Control group were structurally intact and regularly arranged, whereas the MCAO group exhibited severe neuronal damage, including disorganized arrangement, neuronal loss, nuclear pyknosis, hyperchromasia, and vacuolar degeneration. These pathological alterations were alleviated in the treatment groups, particularly in the H-Scu group. Consistently, Nissl staining (Figure 1E) showed normal neuronal morphology and abundant Nissl bodies in the Control group, whereas the MCAO group exhibited reduced neuronal density and marked loss of Nissl bodies. In contrast, neuronal density increased and Nissl bodies were partially restored in the treatment groups. Furthermore, oxidative stress analysis (Figure 1F) showed that Scu extract decreased MDA levels while increasing SOD and GSH-Px activities. Collectively, these results indicate that Scu extract improved neurological function, alleviated brain tissue damage, and mitigated oxidative stress in MCAO rats, suggesting a protective effect against CIRI.

### 3.2. Proteomics Analysis Revealed That Scu Extract Regulated Ferroptosis-Related Proteins and Pathways

**Figure 2:**
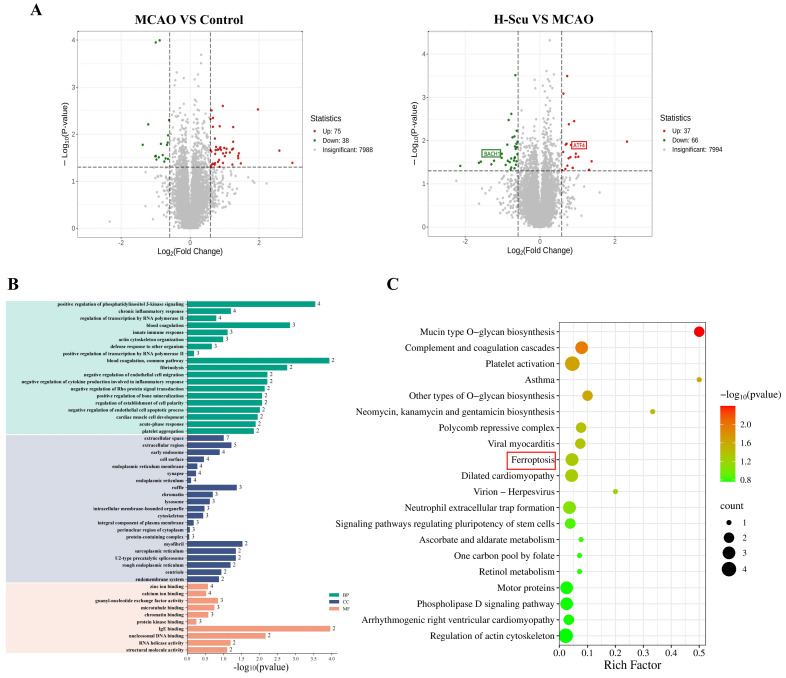
Proteomics Analysis. (**A**) Volcano plot; (**B**) GO enrichment analysis; (**C**) KEGG pathway enrichment analysis. (*n* = 4 for all). Red and green boxes indicated highlighted regions for emphasis.

Subsequently, proteomic analysis was conducted to explore the mechanisms of Scu extract against CIRI. The distribution of differentially expressed proteins (DEPs) was presented in the volcano plot (Figure 2A). MCAO induced 113 DEPs (75 upregulated, 38 downregulated), while H-Scu treatment resulted in 103 DEPs (37 upregulated, 66 downregulated), indicating altered protein expression following CIRI.Among these DEPs, the expression of BACH1, a key regulator of ferroptosis, was significantly downregulated after H-Scu treatment, whereas ATF4, an important regulator of endoplasmic reticulum stress, was significantly upregulated. These findings suggested that Scu extract exerted neuroprotective effects through the regulation of ferroptosis and endoplasmic reticulum stress-related pathways. Furthermore, GO enrichment analysis (Figure 2B) revealed that DEPs regulated by H-Scu were primarily enriched in biological processes such as phagocytosis and innate immune response, suggesting that Scu extract improved the inflammatory microenvironment following CIRI. KEGG pathway enrichment analysis (Figure 2C) further demonstrated significant enrichment of DEPs in the ferroptosis pathway after H-Scu treatment. Collectively, these findings indicate that ferroptosis-related pathways served as key targets through which Scu extract exerted its therapeutic effects against CIRI.

### 3.3. Scu Extract Alleviated Iron Overload and Inhibited Ferroptosis of MCAO Rats

**Figure 3:**
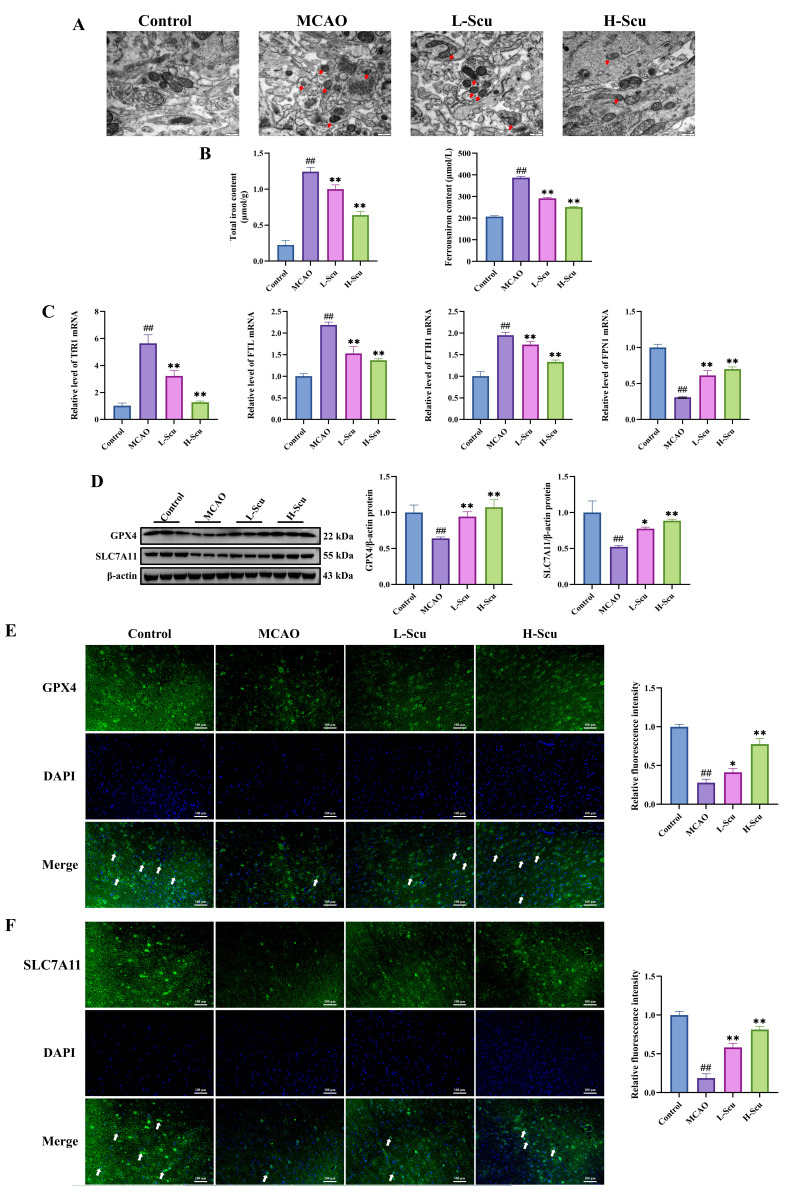
Effects of Scu extract on iron overload and ferroptosis in MCAO rats. (**A**) TEM of cortical tissue (×30,000), The red arrow pointed to damaged mitochondria; (**B**) contents of total iron content and Fe^2+^; (**C**) mRNA expression levels of TFR1, FTH1, FTL, and FPN1; (**D**) representative Western blot bands and quantification of SLC7A11 and GPX4 expressions; (**E**,**F**) representative immunofluorescence images of GPX4 and SLC7A11 (×200) with quantitative analysis of fluorescence intensity, The white arrow pointed to target protein;. (*n* = 3 for all). ^##^ *p* < 0.01 vs. Control group; * *p* < 0.05, ** *p* < 0.01 vs. MCAO group.

This study initially examined mitochondrial morphology, a hallmark phenotype of ferroptosis. As shown in Figure 3A, compared with the Control group, neurons in the MCAO group showed marked swelling, nuclear membrane rupture, and disorganized, fragmented, or even absent mitochondrial cristae, indicating that ferroptosis had occurred in neural cells following CIRI. Notably, these mitochondrial morphological abnormalities were markedly alleviated after Scu extract treatment. Subsequently, indicators of iron metabolism and the ferroptosis defense system were assessed. According to Figure 3B,C, relative to Control group, both total iron and Fe^2+^ levels in the brain tissues of MCAO rats were markedly elevated, indicating pronounced intracellular iron overload following ischemia-reperfusion injury. Consistently, iron metabolism-related gene expression levels were significantly altered. The mRNA expression of TFR1 (iron uptake) and FTH1 and FTL (iron storage), was markedly upregulated, whereas the expression of FPN1 (iron efflux) was markedly downregulated, suggesting that CIRI had disrupted intracellular iron homeostasis. After treatment with Scu extract, these alterations were effectively reversed.

In addition, the ferroptosis-related antioxidant system was further evaluated (Figure 3D–F). Western blot and immunofluorescence analyses showed that the protein expression levels of SLC7A11 and GPX4 were markedly reduced in the MCAO group relative to Control group, indicating suppression of the Xc^−^–GSH–GPX4 antioxidant axis. Scu extract treatment effectively restored SLC7A11 and GPX4 levels. These results further support the proteomic findings, suggesting that Scu extract alleviated CIRI-induced ferroptosis and exerted neuroprotective effects.

### 3.4. UPLC/Q-TOF-MS Identified Four Primary Absorbed Components of Scu Extract

**Figure 4:**
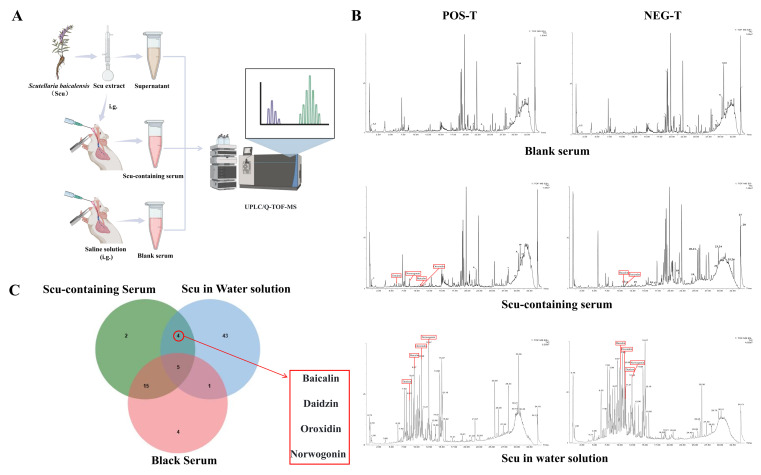
Identification of major absorbed components in Scu extract. (**A**) schematic illustration of sample preparation and UPLC/Q−TOF−MS analysis workflow; (**B**) representative UPLC/Q-TOF-MS chromatograms under positive and negative ion modes; (**C**) Venn diagram.

UPLC/Q-TOF-MS analysis was employed to identify the bioavailable components of Scu extract in the extract, drug-containing serum, and blank serum (Figure 4A). By comparing retention times, accurate masses, and MS/MS fragmentation patterns, and excluding endogenous interference, four prototype compounds absorbed into the bloodstream were identified (Tables S1–S3; Figure 4B,C). The identified compounds were baicalin, daidzin, oroxidin, and norwogonin, indicating that these constituents represented the major bioavailable components of Scu extract following in vivo administration. To further ensure the quality consistency of the extract, HPLC analysis was performed to quantitatively evaluate the major constituents. Baicalin and baicalein were selected as the representative major components for quantification, with contents of 72.78 mg/g and 1.22 mg/g, respectively, determined using the internal standard method. Representative chromatograms and quantitative results are provided in the Supplementary Materials (Figure S1). Based on the identified blood-absorbed components, potential molecular targets were predicted. The SMILES of the four compounds were submitted to SwissTargetPrediction, yielding 147 potential targets, suggesting that Scu extract exerted its effects on CIRI through multiple components and targets.

### 3.5. Network Pharmacology Analysis of Core Targets for Scu Treatment in CIRI

**Figure 5:**
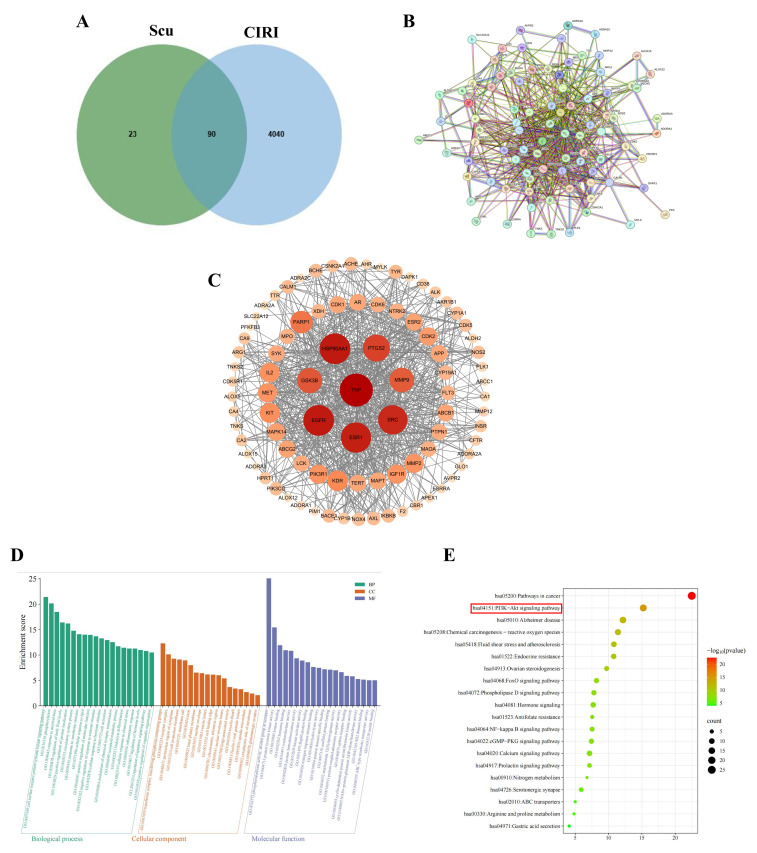
Network pharmacology analysis. (**A**) Venn diagram; (**B**) PPI network; (**C**) Key target screening results; (**D**) GO enrichment analysis; (**E**) KEGG pathway analysis. Red box indicated highlighted regions for emphasis.

To explore the upstream mechanisms of Scu extract, network pharmacology analysis was performed. A total of 147 potential targets and 1500 CIRI-related targets were identified, with 90 overlapping targets (Figure 5A,B). These were used to construct a PPI network, in which TNF, HSP90AA1, and EGFR were identified as hub genes (Figure 5C). Enrichment analyses revealed significant involvement of multiple pathways, particularly the PI3K/AKT signaling pathway (Figure 5D,E), suggesting its key role in mediating the anti-CIRI effects of Scu extract.

### 3.6. Molecular Docking Between Active Compounds and Core Targets

**Figure 6:**
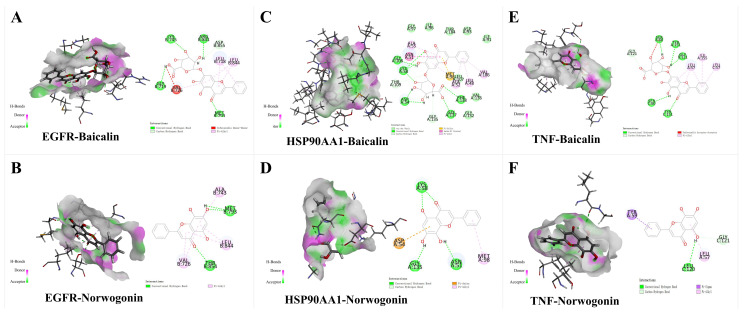
Molecular docking diagrams. (**A**) EGFR docking with Baicalin; (**B**) EGFR docking with Norwogonin; (**C**) HSP90AA1 docking with Baicalin; (**D**) HSP90AA1 docking with Norwogonin; (**E**) TNF docking with Baicalin; (**F**) TNF docking with Norwogonin.

To characterize the interactions between the bioactive constituents of Scu extract and core target proteins, molecular docking was conducted using Discovery Studio 2019. Based on network pharmacology analysis, TNF, HSP90AA1, and EGFR were identified as key targets in the PPI network and selected as receptors. Four major compounds identified by UPLC/Q-TOF-MS, including baicalin, daidzin, oroxidin, and norwogonin, were selected as ligands. The docking results showed that all four compounds bound stably to TNF, HSP90AA1, and EGFR (Figure 6; Table 2), with several ligand–target pairs exhibiting high binding affinity. In molecular docking, lower binding energy indicates stronger predicted binding affinity. Among all tested combinations, norwogonin exhibited the lowest binding energy with TNF (−37.1553 kcal/mol), followed by norwogonin with HSP90AA1 (−32.4806 kcal/mol) and baicalin with EGFR (−30.5804 kcal/mol), suggesting these three combinations have relatively stronger predicted binding interactions. These findings suggest that the absorbed constituents were key mediators of the therapeutic effects of Scu extract on CIRI, likely through interactions with inflammation- and survival-related targets and modulation of the PI3K/AKT pathway and ferroptosis.

### 3.7. Scu Extract Activated PI3K/AKT Pathway Among MCAO Rats

**Figure 7:**
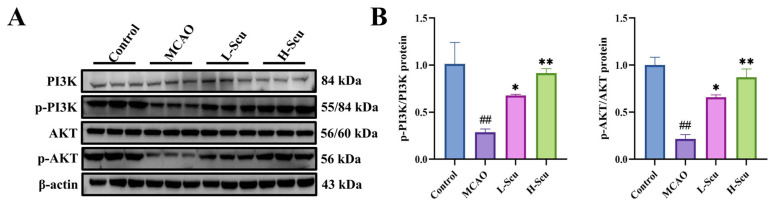
Effects of Scu extract on the PI3K/AKT signaling pathway in MCAO rats. (**A**) protein bands of PI3K, p-PI3K, AKT, and p-AKT; (**B**) relative expression levels of p-PI3K/PI3K and p-AKT/AKT ratios. (*n* = 3 for all). ^##^ *p* < 0.01 vs. Control group; * *p* < 0.05, ** *p* < 0.01 vs. MCAO group.

To validate the key signaling pathways predicted by network pharmacology and molecular docking analyses, the activation status of the PI3K/AKT pathway was further examined. As shown in Figure 7A,B, PI3K and AKT phosphorylation levels were markedly reduced following MCAO, indicating suppression of this pathway after CIRI. Treatment with Scu extract significantly restored these phosphorylation levels, with a more pronounced effect observed in the H-Scu group, suggesting reactivation of PI3K/AKT signaling pathway.

### 3.8. PI3K Suppression Inhibited the Neuroprotection of Scu Extract on MCAO Rats

**Figure 8:**
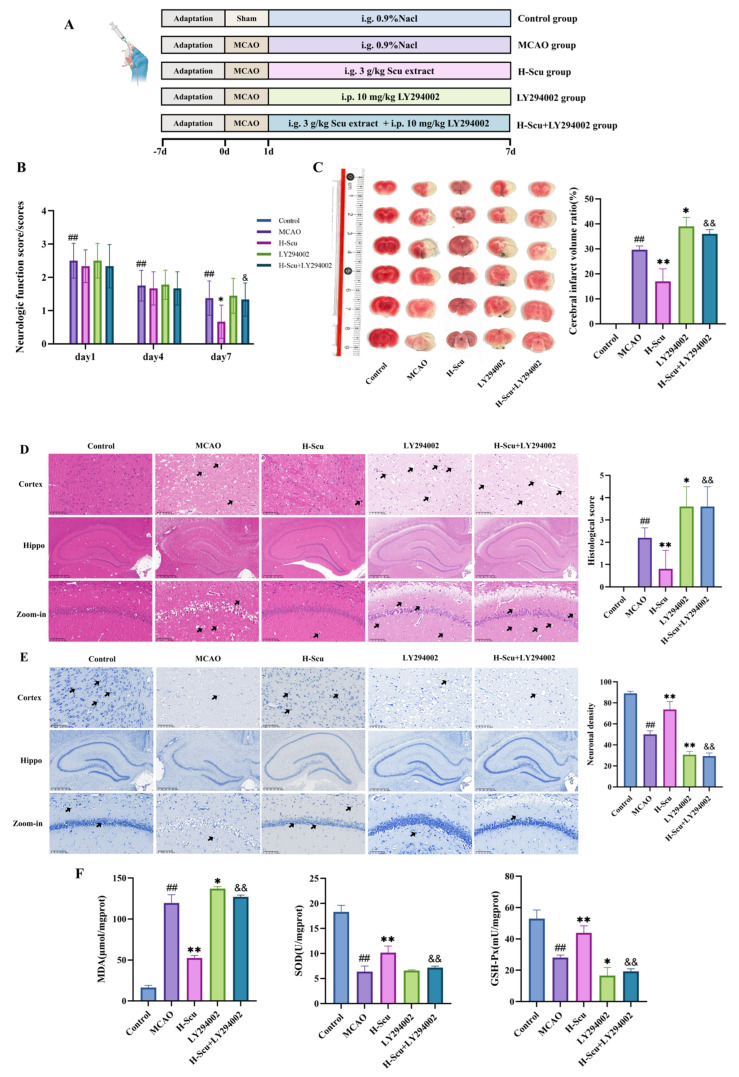
Neuroprotective effects of Scu extract on MCAO rats following PI3K inhibition. (**A**) schematic illustration of model building and intervention; (**B**) neurological function score; (**C**) TTC staining and cerebral infarction volume ratio; (**D**) representative H&E staining images and histological scores (×200), the black arrow pointed to the damaged neuronal cells. (**E**) representative Nissl staining images and neuronal density (×200), the black arrow pointed to the damaged Nissl body. (**F**) MDA content, SOD and GSH-Px activities. (*n* = 12 for (**B**); *n* = 3 for (**C**–**F**)). ^##^ *p* < 0.01 vs. Control group; * *p* < 0.05, ** *p* < 0.01 vs. MCAO group. ^&^ *p* < 0.01 vs. H-Scu group, ^&&^ *p* < 0.01 vs. H-Scu group.

LY294002, a PI3K inhibitor, was used to investigate whether the neuroprotective effects of Scu extract are mediated via the PI3K/AKT pathway (Figure 8A). As shown in Figure 8B–E, compared with the Control group, the MCAO group exhibited significantly increased neurological deficit scores and infarct volume, accompanied by marked histopathological damage, including neuronal disarrangement, cell shrinkage, nuclear pyknosis, reduced Nissl bodies, and decreased neuronal density. Treatment with H-Scu extract markedly reversed these trends. In contrast, LY294002 administration alone further aggravated neurological deficits, infarct volume, and pathological injury. Importantly, co-treatment with H-Scu and LY294002 failed to reverse these detrimental effects. Consistent with these findings, biochemical analysis (Figure 8F) showed that H-Scu extract treatment reversed these oxidative stress changes, whereas LY294002 exacerbated them. Moreover, co-administration of LY294002 abolished the antioxidative effects of H-Scu. Collectively, these results suggest that H-Scu exerts neuroprotective effects against MCAO-induced injury, at least in part, through activation of the PI3K/AKT signaling pathway.

### 3.9. PI3K/AKT Pathway Suppression Weakened Effects of Scu Extract on Ferroptosis in MCAO Rats

**Figure 9:**
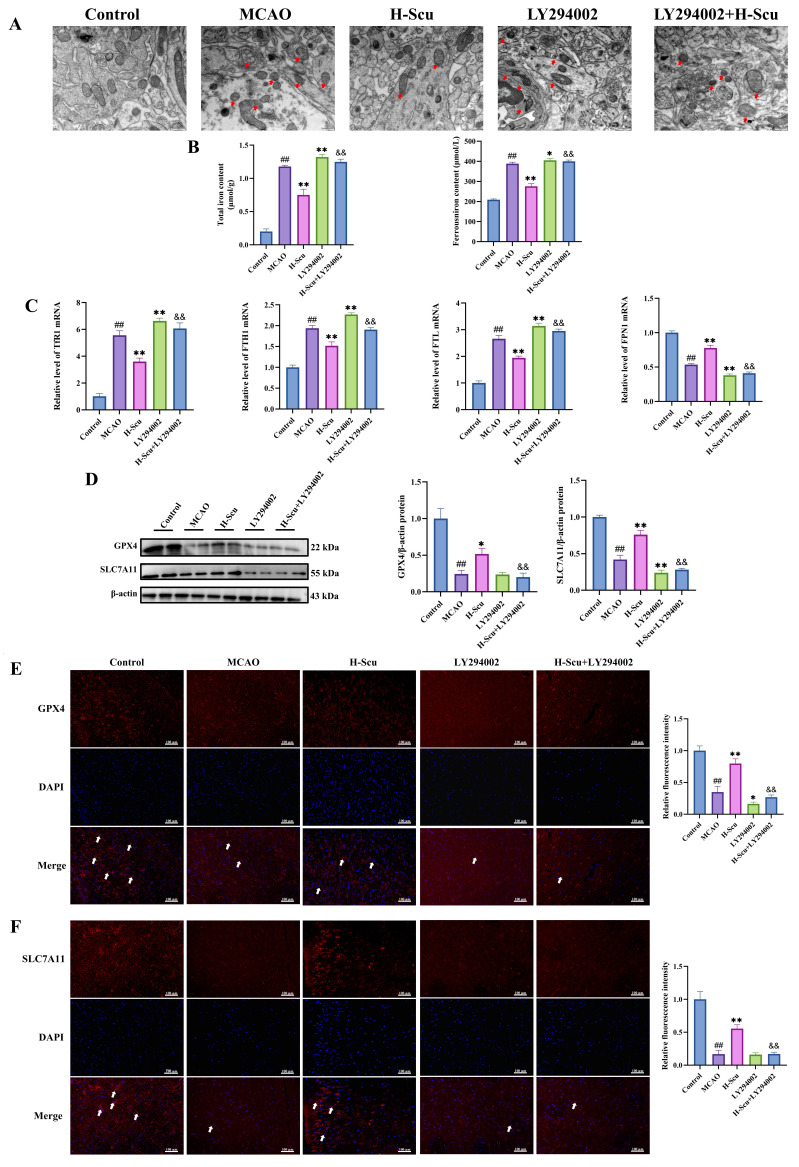
Effects of Scu extract on ferroptosis-related alterations in MCAO rats under PI3K/AKT pathway inhibition. (**A**) TEM of cortical tissue (×30,000), the red arrow pointed to damaged mitochondria; (**B**) contents of total iron content and Fe^2+^; (**C**) mRNA expression levels of TFR1, FTH1, FTL, and FPN1; (**D**) representative Western blot bands and quantification of SLC7A11 and GPX4 expressions; (**E**,**F**) representative immunofluorescence images of GPX4 and SLC7A11 (×200) with quantitative analysis of fluorescence intensity, the white arrow pointed to target protein. (*n* = 3 for all). ^##^ *p* < 0.01 vs. Control group; * *p* < 0.05, ** *p* < 0.01 vs. MCAO group; ^&&^ *p* < 0.01 vs. H-Scu group.

TEM observation revealed that, compared with the Control group, the MCAO group exhibited marked mitochondrial damage, including cristae disruption and even disappearance (Figure 9A). As shown in Figure 9B,C, MCAO rats showed increased total iron content and Fe^2+^ levels, along with upregulated mRNA expression of TFR1, FTH1, and FTL, and downregulated FPN1 expression, indicating disrupted iron homeostasis. Protein expression analysis (Figure 9D) and immunofluorescence staining (Figure 9E,F) showed that SLC7A11 and GPX4 levels were decreased in the MCAO group compared with the Control group. H-Scu treatment ameliorated these ferroptosis-related abnormalities, whereas LY294002 alone further aggravated them, and co-treatment with H-Scu and LY294002 failed to reverse these alterations. Collectively, these results indicate that the inhibitory effect of Scu extract on ferroptosis may be mediated through the PI3K/AKT pathway.

### 3.10. PI3K/AKT Pathway Suppression Abolished Effects of Scu Extract on Ferroptosis in PC12 Cells

**Figure 10:**
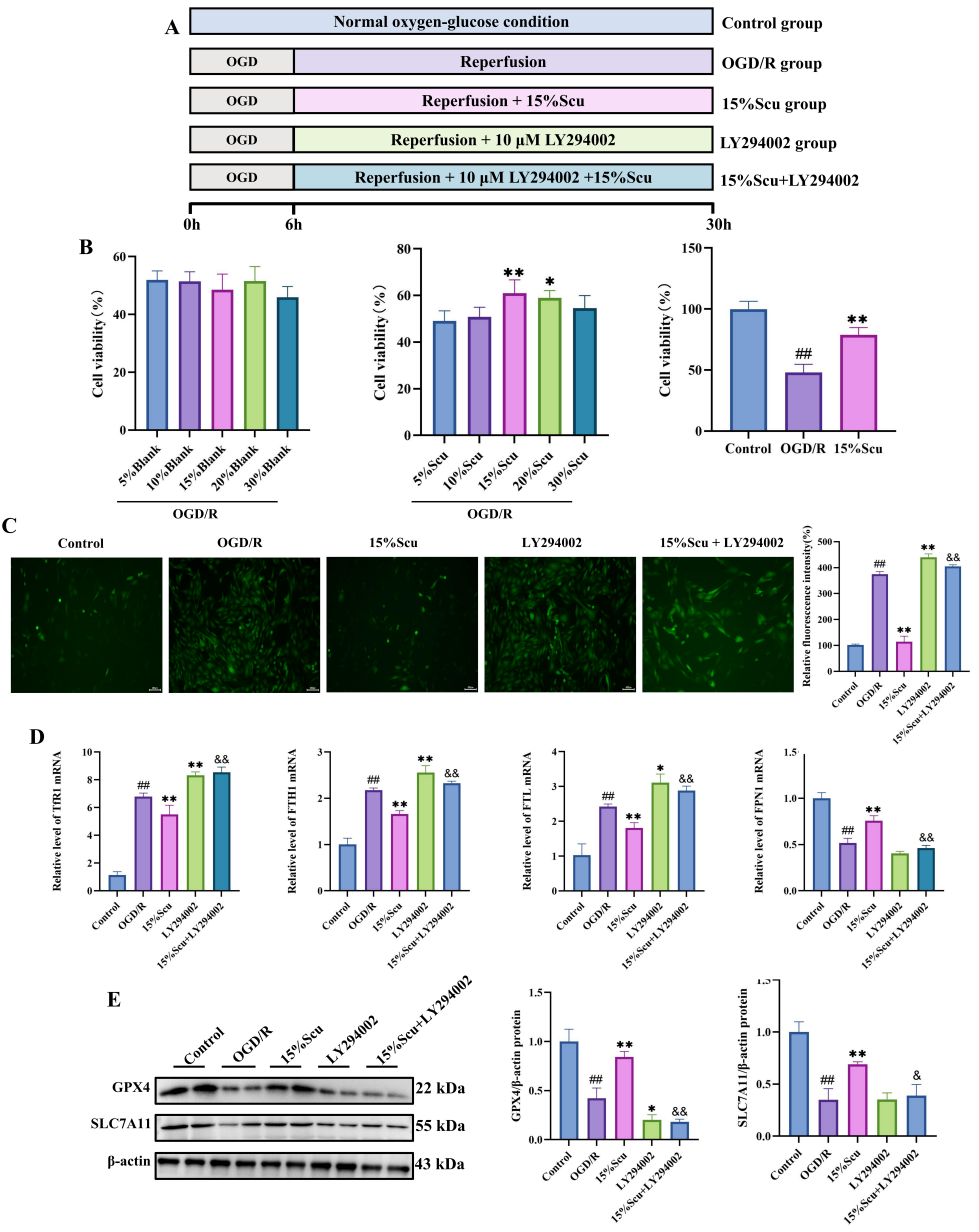
Effects of Scu-containing serum on ferroptosis-related alterations in PC12 cells under PI3K/AKT pathway inhibition. (**A**) schematic illustration of model building and intervention; (**B**) Cell viability assays; (**C**) ROS staining and relative fluorescence intensity; (**D**) mRNA expression levels of TFR1, FTH1, FTL, and FPN1; (**E**) representative Western blot bands and quantification of SLC7A11 and GPX4 expression. (*n* = 6 for (**B**); *n* = 3 for (**C**–**E**)). ^##^ *p* < 0.01 vs. Control group; * *p* < 0.05, ** *p* < 0.01 vs. OGD/R group; ^&^ *p* < 0.01 vs. H-Scu group, ^&&^ *p* < 0.01 vs. 15% Scu group.

To investigate the cellular mechanisms underlying the anti-ferroptotic effects of Scu extract via the PI3K/AKT pathway, an OGD/R-induced PC12 cell model was established (Figure 10A). Under OGD/R conditions, 5–30% blank serum had no significant effect on cell viability, whereas Scu-containing serum increased cell viability in a dose-dependent manner, with the optimal effect observed at 15% (Figure 10B). Subsequently, OGD/R treatment significantly increased the relative fluorescence intensity of ROS and induced ferroptosis-related alterations, including iron accumulation (upregulated expression of TFR1, FTH1, and FTL, and downregulated expression of FPN1) as well as decreased SLC7A11 and GPX4 expression (Figure 10C–E). Importantly, treatment with 15% Scu-containing serum effectively ameliorated these abnormalities. In contrast, treatment with the PI3K inhibitor LY294002 further exacerbated iron dysregulation and suppression of the SLC7A11/GPX4 axis, while co-treatment with Scu-containing serum and LY294002 failed to reverse these changes. Collectively, these results indicate that the PI3K/AKT signaling pathway plays a critical role in mediating the protective and anti-ferroptotic effects of Scu extract in OGD/R-induced PC12 cells.

## 4. Discussion

Following CIRI, interruption of blood flow and subsequent reperfusion trigger a complex pathological cascade, in which disruption of iron metabolism and lipid peroxidation are considered key contributors to neuronal death. Under ischemia–reperfusion conditions, excessive intracellular Fe^2+^ promotes the Fenton reaction, generating large amounts of hydroxyl radicals that attack membrane polyunsaturated fatty acids and initiate lipid peroxidation [37,38]. Mitochondria, as a major source of ROS and a key site of iron metabolism, play a dual role as both initiators of damage and target organelles during CIRI [39]. The accumulation of lipid peroxidation products not only disrupts membrane integrity but also impairs mitochondrial structure and function, leading to abnormal opening of the mitochondrial permeability transition pore, loss of membrane potential, impaired ATP synthesis, and release of apoptotic factors such as cytochrome c, thereby exacerbating neuronal injury [40]. Meanwhile, ischemia–reperfusion impairs antioxidant defense systems, further promoting lipid peroxide accumulation and ultimately resulting in irreversible neuronal injury and death [34]. In this study, Scu extract modulated the expression of ferroptosis-related molecules, as evidenced by significant downregulation of BACH1 and upregulation of ATF4. Previous studies have shown that BACH1 disrupts antioxidant homeostasis by inhibiting the SLC7A11/GPX4 axis, promoting iron accumulation, and activating HO-1 to aggravate lipid peroxidation [41]. In contrast, ATF4 is activated under ischemia–reperfusion stress and has been reported to promote ferroptosis by enhancing lipid peroxidation and inhibiting cystine uptake and GSH synthesis [42]. Notably, given the reported crosstalk between ER stress and the PI3K/AKT pathway in ischemia-reperfusion injury [43], it is possible that Scu extract may modulate both pathways to inhibit ferroptosis. Consistent with these molecular observations, KEGG pathway enrichment analysis revealed that ferroptosis was among the most significantly enriched pathways following Scu intervention, suggesting that it may represent a key target underlying its neuroprotective effects. Furthermore, TEM and biochemical analyses demonstrated that Scu extract alleviated mitochondrial damage and iron overload, while partially restoring iron homeostasis in the brain tissue of MCAO rats. Moreover, Western blot and immunofluorescence analyses showed that Scu extract upregulated SLC7A11 and GPX4 expression, indicating activation of the Xc^−^–GSH–GPX4 antioxidant axis. Collectively, these findings suggest that Scu extract alleviates CIRI-induced neuronal injury by regulating iron metabolism and inhibiting ferroptosis, and this effect is associated with the SLC7A11/GPX4 pathway.

Building upon the ferroptosis-inhibitory effects of Scu extract, this study further explored its pharmacodynamic basis. UPLC/Q-TOF-MS analysis of drug-containing serum identified four bioactive components, namely Baicalin, Daidzin, Oroxidin, and Norwogonin. Previous studies have shown that these compounds possess neuroprotective activities. Specifically, baicalin inhibits ferroptosis by regulating the GPX4/ACSL4/ACSL3 axis [5], while daidzin exhibits antioxidant and anti-apoptotic effects and alleviates mitochondrial dysfunction [44]. In addition, Oroxidin and Norwogonin can scavenge ROS and mitigate oxidative stress-induced neuronal injury [45,46]. To further elucidate the underlying mechanisms, network pharmacology analysis was performed. Integration of compound-related targets with CIRI-associated targets identified 90 overlapping genes, among which TNF, HSP90AA1, and EGFR were identified as hub targets in the PPI network. KEGG pathway enrichment analysis revealed that the PI3K/AKT signaling pathway was significantly enriched, suggesting that it may play a critical role in mediating the anti-CIRI effects of Scu extract.

To further validate the key signaling pathway predicted by network pharmacology and molecular docking analyses, we focused on the PI3K/AKT pathway in subsequent experiments. Notably, PI3K/AKT signaling has been reported to modulate ferroptosis by maintaining redox homeostasis and regulating the SLC7A11/GPX4 axis. Mechanistically, activation of PI3K/AKT inhibits an upstream negative regulatory kinase, thereby allowing a key antioxidant transcription factor to translocate into the nucleus and upregulate the expression of Slc7a11 and Gpx4. In addition, AKT enhances the protein synthesis of SLC7A11 and GPX4 via downstream translational regulators. Consequently, SLC7A11 promotes cystine uptake and GSH production, while GPX4 reduces lipid hydroperoxides, collectively suppressing ferroptosis [16,18]. Although the present study demonstrated in vivo that Scu extract alleviated CIRI in association with inhibition of ferroptosis and activation of the PI3K/AKT pathway, the causal relationship remains to be further validated. In the animal experiments, administration of the PI3K inhibitor LY294002 significantly aggravated neurological deficits, brain tissue damage, and oxidative stress, while weakening the protective effects of Scu extract, suggesting that the PI3K/AKT signaling pathway plays a critical regulatory role in its neuroprotective effects. To further verify this mechanism, in vitro experiments were conducted using an OGD/R-induced PC12 cell model. The results showed that OGD/R induced typical ferroptosis-related alterations, including enhanced oxidative stress, disrupted iron metabolism, and suppression of the SLC7A11/GPX4 axis. Notably, LY294002 further exacerbated these changes, while Scu extract treatment failed to reverse these effects. Collectively, the in vivo and in vitro results consistently indicate that the protective effects of Scu extract against CIRI largely depend on activation of the PI3K/AKT signaling pathway.

Nevertheless, several limitations should be acknowledged. First, although LY294002 provided pharmacological support for the involvement of PI3K/AKT signaling, additional approaches such as genetic intervention would strengthen the mechanistic conclusion. Second, the individual contributions of the four absorbed compounds identified by UPLC/Q-TOF-MS were not separately validated in this study. Third, while network pharmacology and molecular docking offered useful mechanistic clues, the direct interactions between specific compounds and core targets such as TNF, HSP90AA1, and EGFR remain to be experimentally confirmed. Fourth, only male SD rats were used in this study, and the protective effect of Scu extract on CIRI in females was not examined. Considering the established sex differences in pathophysiology, future studies should include both sexes to validate the generalizability of this protective mechanism. Therefore, future studies should further dissect the contributions of individual constituents and clarify the cell-specific signaling events linking PI3K/AKT activation to ferroptosis inhibition in CIRI. In conclusion, this study demonstrates that Scu extract confers significant neuroprotection against CIRI by suppressing ferroptosis. Mechanistically, this effect is closely associated with activation of the PI3K/AKT signaling pathway, as pharmacological inhibition of PI3K largely abolishes its protective actions. These findings not only highlight ferroptosis as a critical therapeutic target in CIRI but also provide a mechanistic basis for the potential application of Scu extract in IS.

## 5. Conclusions

**Figure 11:**
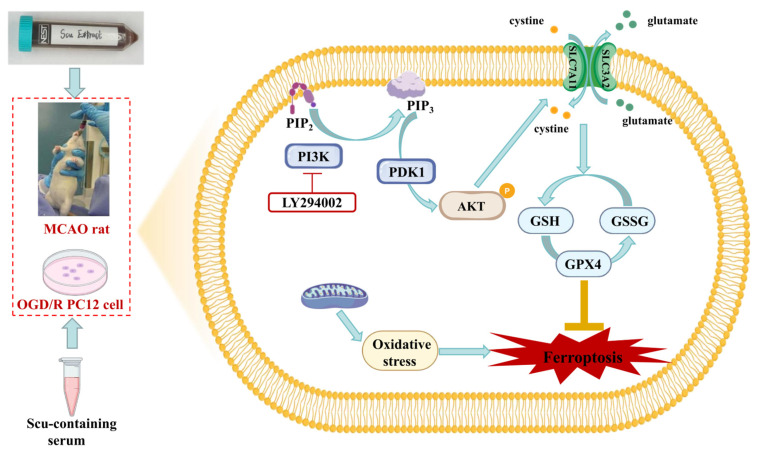
Scu extract regulates and improves CIRI by inhibiting ferroptosis via the PI3K/AKT Pathway.

In summary, this study identifies Scu extract as a potent neuroprotective agent against CIRI, with its effects associated with PI3K/AKT signaling and inhibition of ferroptosis, thereby providing mechanistic insight into its therapeutic potential. (Figure 11).

## Figures and Tables

**Table 1:** qPCR Primers.

Gene	Forward Sequence	Reverse Sequence
*TFR1*	AATGGTTCGTACAGCAGCGGAAG	TAGCACGGAAGTAGTCTCCACGAG
*FTH1*	GCCAGAACTACCACCAGG	CAGTTTCTCAGCATGTTCCC
*FTL*	AGCTGGGCTGAGGAGAGAGA	AGCACCTTCTTCCGGCAGT
*FPN1*	ATGGGTCCTTACTGTCTGCTAC	CCACACTGGACTTCGCAACA

**Table 2:** Binding energy of active compounds with key targets (kcal/mol)).

	EGFR	HSP90AA1	TNF
Baicalin	−30.5804	−24.5119	−25.2794
Daidzin	−12.947	−13.1872	−24.7288
Oroxidin	−24.5743	−19.3261	−26.3358
Norwogonin	−29.8662	−32.4806	−37.1553

## Data Availability

The original contributions presented in this study are included in the article/Supplementary Materials. Further inquiries can be directed to the corresponding authors.

## Supplementary Materials

The following supporting information can be downloaded at: https://www.mdpi.com/article/10.3390/nu18132073/s1, Figure S1: HPLC chromatogram. (A) AM extract sample; (B) Baicalin sample; (C) Baicalein sample. Table S1: Scu in water solution. Table S2: Blank serum. Table S3: Scu-containing serum.
