# Supplementary material for: Scutellaria baicalensis Extract Protects Against Cerebral Ischemia-Reperfusion Injury in Male Rats by Inhibiting Ferroptosis via the PI3K/AKT Pathway
Source: Nutrients. 2026 Jun 24;18(13):2073. doi: 10.3390/nu18132073 (PMC13363625; doi:10.3390/nu18132073)
Supplement: Supplementary file 1 [file nutrients-18-02073-s001.zip › nutrients-4331704-supplementary.pdf]

Supplementary material

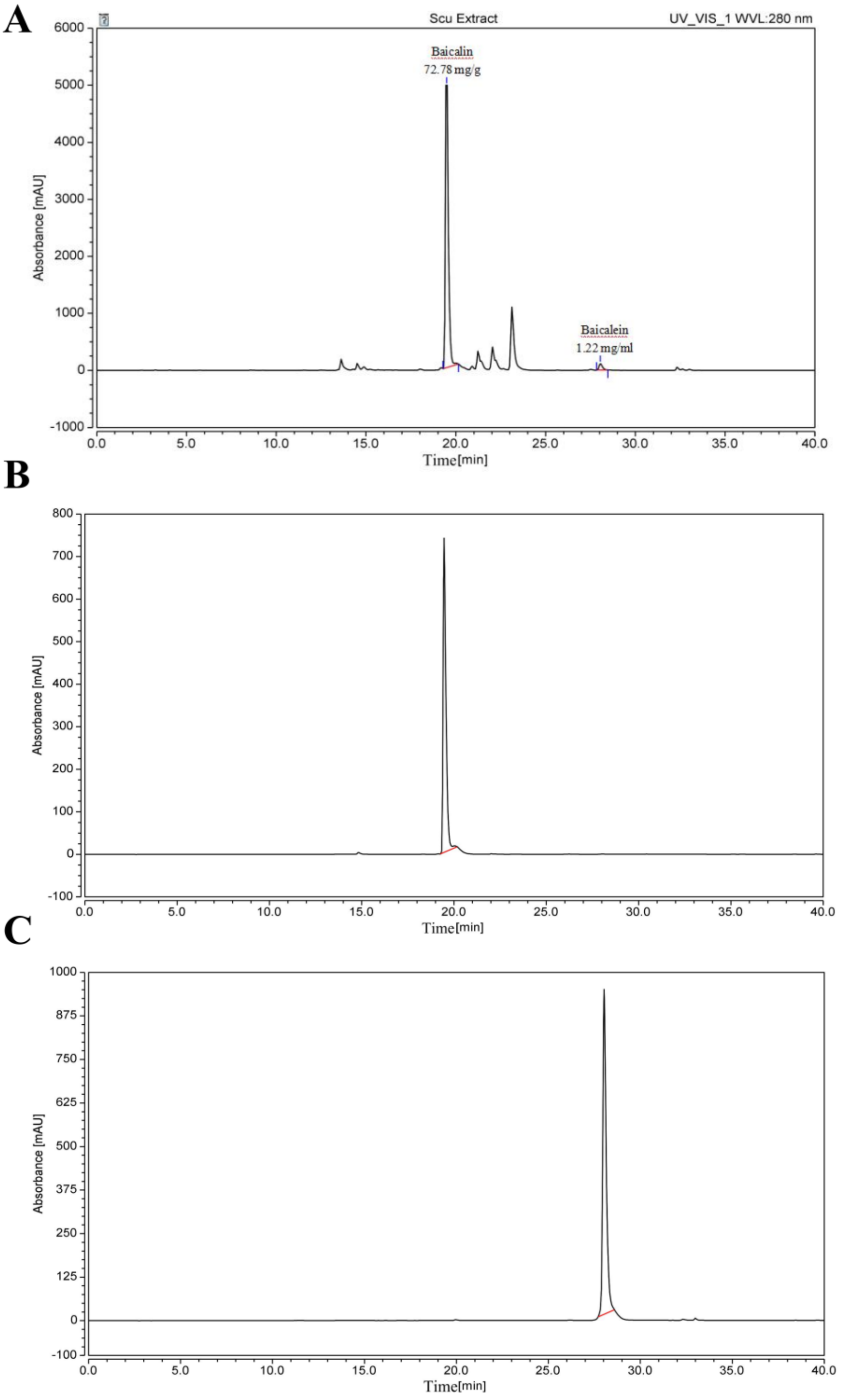

Figure S1. HPLC chromatogram. (A) AM extract sample; (B) Baicalin sample; (C) Baicalein sample.

Table S1. Scu in water solution.

No	Component name	Formula	Identification status	Observed m/z	Observed RT (min)	Adducts
1	Adenine nucleoside	C10H13N5O4	Identified	268.1042	1.09	+H
2	Viscidulin I	C15H10O7	Identified	303.05	7.45	+H, +Na
3	Salidroside	C14H20O7	Identified	339.0864	7.61	+K
4	Epiberberine	C20H18NO4	Identified	375.0861	7.62	+K
5	2,6,2',4'-Tetrahydroxy-6'-methoxychaleone	C16H14O6	Identified	303.086	7.75	+H, +Na
6	Phthalate	C8H6O4	Identified	167.0341	7.76	+H
7	Scutellarin	C21H18O12	Identified	463.0871	7.79	+H, +Na
8	Daidzin	C21H20O9	Identified	417.118	8.61	+H
9	5,7,2',6'-Tetrahydroxyflavone	C15H10O6	Identified	287.0551	8.94	+H
10	Baicalin	C21H18O11	Identified	447.0922	9.57	+H, +Na, +K
11	5,7,2',5'-Tetrahydroxy-8,6'-dimethoxy flavone	C17H14O8	Identified	347.076	9.58	+H
12	Isoscutellarein	C15H10O6	Identified	287.0552	9.62	+H
13	Cosmetin	C21H20O10	Identified	433.1129	9.63	+H, +Na, +K
14	Neochlorogenic acid	C16H18O9	Identified	377.0867	9.7	+Na
15	Carthamidin	C15H12O6	Identified	289.0707	9.74	+H
16	Eriodictyol	C15H12O6	Identified	289.0705	9.91	+H
17	Apigenin	C15H10O5	Identified	271.0602	9.91	+H
18	Dihydrobaicalin	C21H20O11	Identified	449.1082	10.02	+H, +Na
19	Dihydrobaicalein	C15H12O5	Identified	273.0757	10.02	+H
20	5,7,4'-Trihydroxy-8-methox	C16H14O6	Identified	303.086	10.25	+H

	yflavanone					
21	Acacetin	C16H12O5	Identified	285.0754	10.33	+H
22	7-Ethyl-10-hydroxycamptothecin	C22H20N2O5	Identified	431.0971	10.57	+K
23	Oroxindin	C22H20O11	Identified	461.1075	10.99	+H, +Na, +K
24	Dihydrooroxylin A	C16H14O5	Identified	287.0907	11.11	+H
25	Salvigenin	C18H16O6	Identified	329.1017	12.35	+H
26	Scutevulin	C16H12O6	Identified	301.0705	12.35	+H, +Na
27	Viscidulin II	C17H14O7	Identified	331.081	12.4	+H, +Na
28	Norwogonin	C15H10O5	Identified	271.0601	12.47	+H, +Na
29	Rivularin	C18H16O7	Identified	345.0965	14.51	+H, +Na, +K
30	Wogonin	C16H12O5	Identified	285.0759	14.67	+H, +Na
31	Chrysin	C15H10O4	Identified	255.0652	14.76	+H
32	5,8-Dihydroxy-6,7-dimethoxyflavone	C17H14O6	Identified	315.0863	14.94	+H, +Na, +K
33	Neobaicalein	C19H18O8	Identified	375.1074	15.08	+H, +Na, +K
34	Panicolin	C17H14O6	Identified	315.0862	15.29	+H, +Na
35	5,2'-Dihydroxy-6,7,8-trimethoxyflavone	C18H16O7	Identified	345.0963	15.83	+H, +Na, +K
36	Coptisine	C19H14NO4	Identified	343.0818	16.77	+Na
37	5-Hydroxy-7,8-dimethoxyflavone	C17H14O5	Identified	299.0917	17.6	+H
38	13-Tetradecenyl acetate	C16H30O2	Identified	277.2161	20.57	+Na
39	Methylcinnamate	C10H10O2	Identified	163.0754	21.03	+H
40	Methyl isoheptadecanoate	C18H36O2	Identified	285.2789	28.33	+H
41	Camptothecin	C20H16N2O4	Identified	371.1015	30.28	+Na

42	2-[3,5-Dihydroxy-2-[2-(3-hydroxy-4-methoxyphenyl)ethoxy]-6-(hydroxymethyl)oxan-4-yl]oxy-6-methyloxane-3,4,5-triol	C21H32O12	Identified	475.1829	5.77	-H, +HCOO
43	Cianidanol	C15H14O6	Identified	289.0713	6.09	-H
44	Mipax	C10H10O4	Identified	239.0555	7.09	+HCOO
45	Viscidulin I	C15H10O7	Identified	301.0354	7.44	-H
46	Isoscutellarein	C15H10O6	Identified	285.0407	7.79	-H
47	Scutellarin	C21H18O12	Identified	461.0735	7.79	-H
48	Hormothamnione	C21H20O8	Identified	445.1128	8.43	+HCOO
49	Syringaresinol	C22H26O8	Identified	417.1549	8.5	-H
50	5,7,2',6'-Tetrahydroxyflavone	C15H10O6	Identified	285.0406	8.94	-H
51	5,7,4'-Trihydroxy-8-methoxyflavanone	C16H14O6	Identified	301.0719	9.14	-H
52	Eriodictyol	C15H12O6	Identified	287.0561	9.42	-H
53	5,7,2',5'-Tetrahydroxy-8,6'-dimethoxy flavone	C17H14O8	Identified	345.0616	9.6	-H
54	Cosmetin	C21H20O10	Identified	431.0985	9.63	-H
55	Carthamidin	C15H12O6	Identified	287.056	9.74	-H
56	2,6,2',4'-Tetrahydroxy-6'-methoxychaleone	C16H14O6	Identified	347.0768	9.97	+HCOO
57	Isomartynoside	C31H40O15	Identified	651.2307	9.97	-H, +HCOO
58	Dihydrobaicalin	C21H20O11	Identified	447.0936	10.03	-H
59	Baicalin	C21H18O11	Identified	445.0787	10.17	-H
60	Acacetin	C16H12O5	Identified	283.0613	10.32	-H
61	Alpinetin	C16H14O4	Identified	315.0869	10.93	+HCOO

62	Oroxindin	C22H20O11	Identified	459.0933	10.99	-H
63	Dihydrooroxylin A	C16H14O5	Identified	285.0761	11.11	-H
64	Daidzin	C21H20O9	Identified	461.1084	11.11	+HCOO
65	Panicolin	C17H14O6	Identified	313.0715	11.21	-H
66	Scutevulin	C16H12O6	Identified	299.0559	12.35	-H
67	Norwogonin	C15H10O5	Identified	269.0454	12.47	-H
68	Apigenin	C15H10O5	Identified	269.0457	12.63	-H
69	Dihydrobaicalein	C15H12O5	Identified	271.061	12.63	-H
70	Viscidulin II	C17H14O7	Identified	329.0669	12.74	-H
71	Rivularin	C18H16O7	Identified	389.0877	13.62	+HCOO
72	Wogonin	C16H12O5	Identified	283.0615	14.67	-H
73	Chrysin	C15H10O4	Identified	253.0508	14.75	-H
74	Salvigenin	C18H16O6	Identified	373.0928	14.86	+HCOO
75	Skullcapflavone II	C19H18O8	Identified	373.0931	15.08	-H
76	5,8-Dihydroxy-6,7-dimethoxyflavone	C17H14O6	Identified	313.0715	15.29	-H
77	5,2'-Dihydroxy-6,7,8-trimethoxyflavone	C18H16O7	Identified	343.0825	15.83	-H
78	Methyl palmitelaidate	C17H32O2	Identified	313.2385	17.23	+HCOO
79	Tributyl phosphate	C12H27O4P	Identified	311.1655	21.69	+HCOO
80	13-Tetradecenyl acetate	C16H30O2	Identified	253.2173	25.54	-H
81	Diisobutyl succinate	C12H22O4	Identified	275.1503	26.38	+HCOO
82	Methyl isoheptadecanoate	C18H36O2	Identified	283.2641	29.73	-H
83	Maxacalcitol	C26H42O4	Identified	463.3102	33.36	+HCOO

Table S2. Blank serum.

No	Component name	Formula	Identification status	Observed m/z	Observed RT (min)	Adducts
1	Benzoic acid	C7H6O2	Identified	123.044	1.09	+H
2	p-Coumaric acid	C9H8O3	Identified	165.0545	1.09	+H
3	Methylcinnamate	C10H10O2	Identified	163.0753	21	+H
4	Methyl palmitelaidate	C17H32O2	Identified	291.2321	23.82	+Na
5	Methyl isoheptadecanoate	C18H36O2	Identified	285.2793	28.31	+H
6	Methyl myristate	C15H30O2	Identified	243.2317	28.83	+H
7	Methyl (E)-octadec-2-enoate	C19H36O2	Identified	319.2636	29.42	+Na
8	Camptothecin	C20H16N2O4	Identified	371.1022	30.25	+Na
9	Calcium valproate	C16H30CaO4	Identified	365.1377	30.58	+K
10	bis[(2R)-2-Ethylhexyl] benzene-1,2-dicarboxylate	C24H38O4	Identified	413.2672	30.59	+Na, +H, +K
11	Methyl palmitate	C17H34O2	Identified	271.2638	31.07	+H
12	Eugenol	C10H12O2	Identified	165.0913	31.96	+H
13	Rivularin	C18H16O7	Identified	343.0857	9.47	-H
14	Methyl 9-oxononanoate_1	C10H18O3	Identified	185.1181	13.86	-H
15	Diisooctyl phthalate_1	C24H38O4	Identified	389.2693	16.18	-H, +HCOO
16	Methyl palmitelaidate	C17H32O2	Identified	313.2378	17.2	+HCOO
17	Baicalein	C15H10O5	Identified	315.0496	18.45	+HCOO
18	13-Tetradecenyl acetate	C16H30O2	Identified	253.2174	25.19	-H
19	Methyl myristate_1	C15H30O2	Identified	241.2175	25.52	-H
20	Eicosane	C20H42	Identified	327.3278	25.55	+HCOO
21	11,13-Eicosadienoic acid, methyl ester	C21H38O2	Identified	367.285	27.03	+HCOO
22	Methyl palmitate	C17H34O2	Identified	269.2481	28.29	-H
23	Methyl isoheptadecanoate	C18H36O2	Identified	283.264	29.71	-H

24	Methyl (Z)-icos-11-enoate	C21H40O2	Identified	369.301	29.88	+HCOO
25	Methyl nonadecanoate_1	C20H40O2	Identified	311.2948	31.74	-H
26	Methyl tricosanoate_1	C24H48O2	Identified	367.3576	31.81	-H
27	Methyl myristate_1	C15H30O2	Identified	241.2171	32.8	-H
28	Methyl tetracosanoate	C25H50O2	Identified	381.3737	33.2	-H
29	Methyl heneicosanoate	C22H44O2		339.327	33.47	-H

Table S3. Scu-containing serum.

No	Component name	Formula	Identification status	Observed m/z	Observed RT (min)	Adducts
1	p-Coumaric acid	C9H8O3	Identified	165.0546	1.09	+H
2	Daidzin	C21H20O9	Identified	417.1188	5.97	+H
3	Norwogonin	C15H10O5	Identified	271.0603	8.55	+H
4	Baicalin	C21H18O11	Identified	447.0931	10.8	+H
5	Oroxindin	C22H20O11	Identified	461.1093	10.97	+H
6	Methylcinnamate	C10H10O2	Identified	163.0753	21	+H
7	Methyl isoheptadecanoate	C18H36O2	Identified	285.2792	28.3	+H
8	Methyl (E)-octadec-2-enoate	C19H36O2	Identified	319.2634	29.42	+Na
9	Camptothecin	C20H16N2O4	Identified	371.1018	30.25	+Na
10	bis[(2R)-2-Ethylhexyl] benzene-1,2-dicarboxylate	C24H38O4	Identified	413.2671	30.59	+Na, +H
11	Methyl palmitate	C17H34O2	Identified	271.2637	31.07	+H
12	Eugenol	C10H12O2	Identified	165.0912	31.3	+H
13	Baicalin	C21H18O11	Identified	445.0764	10.81	-H
14	Oroxindin	C22H20O11	Identified	459.0957	10.98	-H
15	Methyl 9-oxononanoate	C10H18O3	Identified	185.1177	13.85	-H
16	Diisooctyl phthalate	C24H38O4	Identified	389.2695	16.18	-H, +HCOO
17	Methyl palmitelaidate	C17H32O2	Identified	313.2386	17.2	+HCOO
18	Baicalein	C15H10O5	Identified	315.0494	21.56	+HCOO
19	13-Tetradecenyl acetate	C16H30O2	Identified	253.2178	25.19	-H
20	Methyl myristate	C15H30O2	Identified	241.2169	25.52	-H
21	Eicosane	C20H42	Identified	327.3275	25.55	+HCOO
22	Methyl palmitate	C17H34O2	Identified	269.2484	28.29	-H
23	Methyl isoheptadecanoate	C18H36O2	Identified	283.2642	29.71	-H
24	Methyl (Z)-icos-11-enoate	C21H40O2	Identified	369.3011	29.88	+HCOO

25	Methyl nonadecanoate	C20H40O2	Identified	311.2953	31.74	-H
26	Methyl tricosanoate	C24H48O2	Identified	367.3579	31.81	-H
27	Methyl tetracosanoate	C25H50O2	Identified	381.3739	33.18	-H
28	Methyl heneicosanoate	C22H44O2	Identified	339.3265	33.47	-H
